# Regulation of BDNF transcription by Nrf2 and MeCP2 ameliorates MPTP-induced neurotoxicity

**DOI:** 10.1038/s41420-022-01063-9

**Published:** 2022-05-20

**Authors:** Qianqian Cao, Qiuming Zou, Xin Zhao, Yimin Zhang, Youge Qu, Nanbu Wang, Shigeo Murayama, Qi Qi, Kenji Hashimoto, Song Lin, Ji-chun Zhang

**Affiliations:** 1 Department of Physiology, School of Medicine, Jinan University, 510632 Guangzhou, China; 2 Department of Nuclear Medicine, The First Affiliated Hospital of Jinan University, Guangzhou, China; 3 Department of Pharmacology, School of Medicine, Jinan University, 510632 Guangzhou, China; 4 Division of Clinical Neuroscience, Chiba University Center for Forensic Mental Health, Chiba, 260-8670 Japan; 5 The First Affiliated Hospital of Guangzhou University of Chinese Medicine, 510632 Guangzhou, China; 6 Department of Neuropathology (Brain Bank for Aging Research), Tokyo Metropolitan Geriatric Hospital & Institute of Gerontology, Tokyo, 173-0015 Japan

**Keywords:** Parkinson's disease, Neurodegeneration

## Abstract

Mounting evidence suggests the key role of brain-derived neurotrophic factor (BDNF) in the dopaminergic neurotoxicity of Parkinson’s disease (PD). Activation of NF-E2-related factor-2 (Nrf2) and inhibition of methyl CpG-binding protein 2 (MeCP2) can regulate BDNF upregulation. However, the regulation of BDNF by Nrf2 and MeCP2 in the PD pathogenesis has not been reported. Here, we revealed that Nrf2/MeCP2 coordinately regulated BDNF transcription, reversing the decreased levels of BDNF expression in 1-methyl-4-phenylpyridinium (MPP^+^)-treated SH-SY5Y cells and 1-methyl-4-phenyl-1,2,3,6-tetrahydropyridine (MPTP)-treated mice. Repeated administration of sulforaphane (SFN, an Nrf2 activator) attenuated dopaminergic neurotoxicity in MPTP-treated mice through activation of BDNF and suppression of MeCP2 expression. Furthermore, intracerebroventricular injection of MeCP2-HDO, a DNA/RNA heteroduplex oligonucleotide (HDO) silencing MeCP2 expression, ameliorated dopaminergic neurotoxicity in MPTP-treated mice via activation of Nrf2 and BDNF expression. Moreover, we found decreased levels of Nrf2 and BDNF, and increased levels of MeCP2 protein expression in the striatum of patients with dementia with Lewy bodies (DLB). Interesting, there were correlations between BDNF and Nrf2 (or MeCP2) expression in the striatum from DLB patients. Therefore, it is likely that the activation of BDNF transcription by activation of Nrf2 and/or suppression of MeCP2 could be a new therapeutic approach for PD.

## Introduction

Parkinson’s disease (PD) is a progressive neurodegenerative disorder that affects movement. The pathogenesis of PD is described by the selective degeneration of dopaminergic neurons in the substantia nigra pars compacta (SNc) of the brain [1]. Although both the genetic and environmental factors play a role in the etiology of PD [2], the precise molecular mechanisms underlying the etiology of PD remain poorly understood. Brain-derived neurotrophic factor (BDNF), a member of the neurotrophin family [3–5], and its receptor TrkB are highly expressed in the neocortex, hippocampus, striatum, and brainstem [3–6]. Interestingly, it is reported that BDNF co-localizes with dopaminergic neurons in the SNc, and that BDNF could promote dopaminergic neuronal survival [7, 8]. Clinical studies showed that BDNF levels are decreased in PD patients, suggesting that reduced levels of BDNF may be involved in the pathogenesis of PD [3, 9, 10]. Induction of BDNF in either the striatum or the midbrain by transplanting of modified fibroblasts protected against 6-hydroxydopamine (6-OHDA) or 1-methyl-4-phenyl-1,2,3,6-tetrahydropyridine (MPTP)-induced nigrostriatal degeneration [11]. In addition, BDNF could ameliorate the rotational behavioral deficit and increases the turnover of dopamine in the striatum by regulating dopaminergic neurotransmission [12]. Adeno-associated virus (AAV)-mediated BDNF overexpression attenuated 6-OHDA-induced striatal medium spiny neuronal lesions [13]. Collectively, it is likely that BDNF plays important roles in the survival of dopaminergic neurons and dopaminergic neurotoxicity in the SNc which are implicated in the pathogenesis of PD.

Sulforaphane (SFN: 1-isothiocyanato-4-methylsulfinylbutane), an organosulfur compound, is identified as a potent activator of the transcription factor NF-E2-related factor-2 (Nrf2) that regulates antioxidant and anti-inflammatory responses [14–17]. Mounting evidence shows that SFN could exert potent beneficial effects in various animal models such as depression and schizophrenia [14, 18–22]. It is suggested that the beneficial effects of SFN could be due to potent neuroprotective effects via upregulation of BDNF expression [15, 19, 21, 22]. Methyl CpG-binding protein 2 (MeCP2) is a transcription repressor, which blocks BDNF transcription [23, 24]. Recently, it is reported that activation of Nrf2 by SFN increases BDNF expression and decreases MeCP2 expression, resulting in antidepressant-like actions in rodents [25]. Moreover, several findings show the connection of Nrf2 with PD. First, Nrf2 activity declines with age. Second, the transcriptional activity of Nrf2 can be restored pharmacologically in old animals. Third, in nigral dopaminergic neurons, Nrf2 is located at the cytosol, whereas in age-matched patients with PD, Nrf2 is found at the nucleus, suggesting an attempt to reduce oxidative stress through Nrf2-dependent transcription of antioxidant enzymes [26]. In addition, it is shown that patients with mutations in the MeCP2 exhibit motor deficits similar to PD patients [27, 28]. These evidence suggest that both Nrf2 and MeCP2 might be involved in the pathogenesis of PD. However, the role of Nrf2 and MeCP2 in regulating of BDNF transcription in the pathogenesis of PD has not been reported. Therefore, it is interesting to investigate the role of BDNF transcription in PD models by Nrf2 and MeCP2 regulation.

This study was undertaken to investigate whether activation of BDNF by Nrf2 and MeCP2 can affect dopaminergic neurotoxicity using in vitro and in vivo models of PD. First, we examined whether activation of Nrf2 by SFN could ameliorate 1-methyl-4-phenylpyridinium (MPP^+^)-induced neurotoxicity in SH-SY5Y cells through activation of BDNF and inhibition of MeCP2. Second, we examined the role of Nrf2, BDNF and MeCP2 in the dopaminergic neurotoxicity of SNc after repeated MPTP administration. Third, using the DNA/RNA heteroduplex oligonucleotide (HDO), a newly developed technology for gene silencing [29, 30], we examined the role of MeCP2 in the regulation of BDNF expression and MPTP-induced neurotoxicity. Finally, we measured the expression of Nrf2, BDNF, and MeCP2 in the striatum from dementia with Lewy bodies (DLB) patients.

## Results

### Effects of SFN on BDNF transcription in vitro

**Fig. 1:**
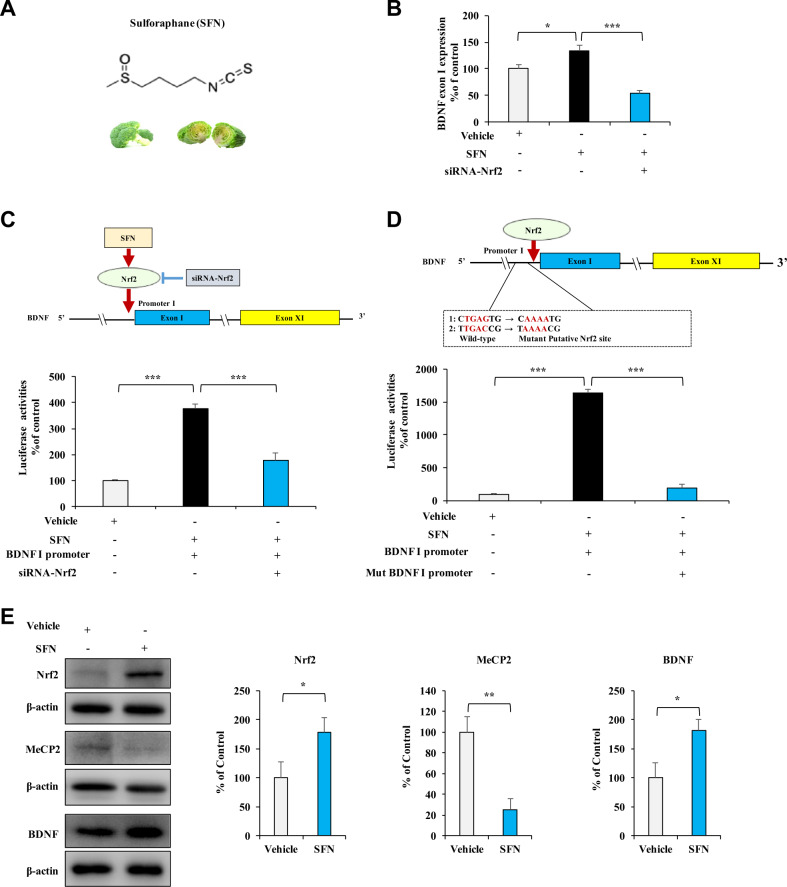
Effects of SFN on BDNF transcription in vitro. **A** Chemical structure of SFN. **B** SFN enhanced *Bdnf* exon I expression via Nrf2 activation. Following treatment with SFN or/and Nrf2 siRNA, SH-SY5Y cells were collected and analyzed by real-time PCR for *Bdnf* exon I expression. Data are shown as mean ± SEM (*n* = 4). *P < 0.05; ***P < 0.001 (one-way ANOVA: *F*_2,11_ = 76.90, *P* < 0.001). **C** Nrf2 knocking down abolished the activation of *Bdnf* exon I promoter in HEK293T cells. HEK293T cells were treated with SFN or/and siRNA, followed by luciferase reporter analysis. Data are shown as mean ± SEM (*n* = 4). ***P < 0.001 (one-way ANOVA: *F*_2,11_ = 29.579, *P* < 0.001). **D** Mutations of the Nrf2-binding motif in *Bdnf* exon I promoter blocked BDNF transcription. HEK293T cells were treated with SFN in the presence of wild-type or mutated *Bdnf* exon I promoter followed by luciferase reporter analysis. Data are shown as mean ± SEM (*n* = 4). ***P < 0.001 (one-way ANOVA: *F*_2,11_ = 198.666, *P* < 0.001). **E** Effect of SFN on Nrf2, MeCP2, and BDNF expression in SH-SY5Y cells. Cells were treated with SFN (1 µM) for 24 h, followed by western blotting analysis with Nrf2, MeCP2, and BDNF antibody. β-actin levels were used as a loading control. Data are shown as mean ± SEM (*n* = 5). *P < 0.05 (Student *t* test: *T* = −2.365, *P* = 0.046 for Nrf2, *T* = 4.082, *P* = 0.004 for MeCP2, and *T* = −2.745, *P* = 0.025 for BDNF).

Based on Nrf2-binding characteristics [31, 32], we reported the *Bdnf* exon I promoter as two potential Nrf2 consensus binding motifs [25]. The real-time PCR assay indicated that SFN upregulated *Bdnf* exon I mRNA expression, which was abolished by Nrf2 knocking down (Fig. 1A, B). To further determine the role of SFN on Nrf2-induced BDNF transcription, we used siRNA-Nrf2 and mutated the potential consensus binding motifs of Nrf2 in the *Bdnf* exon I promoter, followed by luciferase reporter assay. SFN prominently activated the *Bdnf* exon I promoter, which could be reversed by siRNA-Nrf2 or the mutation in the *Bdnf* exon I promoter (Fig. 1C, D). The western blots analysis showed that SFN significantly increased the levels of Nrf2 and BDNF proteins, and decreased MeCP2 protein in SH-SY5Y cells (Fig. 1E). These data demonstrate that SFN directly activates BDNF transcription through Nrf2 binding with the *Bdnf* exon I promoter, resulting in an increased expression of BDNF protein.

### Effects of SFN on MPP^+^-induced alterations in the expression of Nrf2, MeCP2, and BDNF in SH-SY5Y cells

**Fig. 2:**
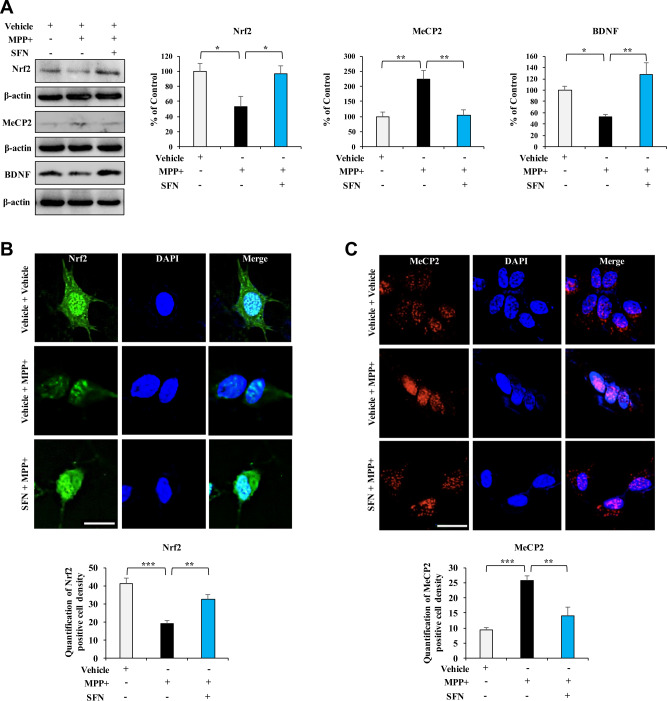
Effects of SFN on MPP^+^-induced downregulation of BDNF in SH-SY5Y cells. **A** Effects of SFN on the expressions of Nrf2, MeCP2, and BDNF in MPP^+^-treated SH-SY5Y cells. Cells were treated with SFN (1 μM) and/or MPP^+^ (1 mM) 24 h, followed by western blotting assay. Data are shown as mean ± SEM (*n* = 4). *P < 0.05, **P < 0.01 (one-way ANOVA: Nrf2: *F*_2,11_ = 5.266, *P* = 0.031, MeCP2: *F*_2,11_ = 10.110, *P* = 0.005, BDNF: *F*_2,11_ = 8.191, *P* = 0.009). **B**, **C** The immunofluorescence staining for Nrf2 and MeCP2 in SH-SY5Y cells treated with SFN (1 μM) and/or MPP^+^ (1 mM) treated for 24 h. Scale bar = 50 μm. There were statistical differences among the three groups (one-way ANOVA, Nrf2: *F*_2,11_ = 17.897, *P* = 0.001, MeCP2: *F*_2,11_ = 19.030, *P* = 0.001).

It is reported that MPP^+^ is the active neurotoxic compound, and that it selectively taken up into dopaminergic neurons to kill the dopaminergic neurons [33]. Following treatment with MPP^+^, the expressions of Nrf2 and BDNF were significantly decreased in SH-SY5Y cells, whereas the expressions of MeCP2 were significantly increased by MPP^+^ treatment (Fig. 2A). Furthermore, SFN significantly ameliorated the downregulation of Nrf2 and BDNF, and the upregulation of MeCP2 in SH-SY5Y cells by MPP^+^ treatment (Fig. 2A).

Since both Nrf2 and MeCP2 function in the nucleus as a transcription regulator, cellular localization of the two proteins were examined by immunofluorescence staining. Data showed that MPP^+^ notably decreased Nrf2 expression in the nucleus, which could be reversed by SFN treatment (Fig. 2B). Moreover, treatment of MPP^+^ caused the nuclear localization of MeCP2, which could be reversed by SFN (Fig. 2C). The results suggest that SFN reverses MPP^+^-induced alterations in the expression of Nrf2, MeCP2, and BDNF in SH-SY5Y cells.

### Effects of SFN on dopaminergic neurotoxicity associated with Nrf2/MeCP2-induced BDNF transcription in MPTP-treated mice

**Fig. 3:**
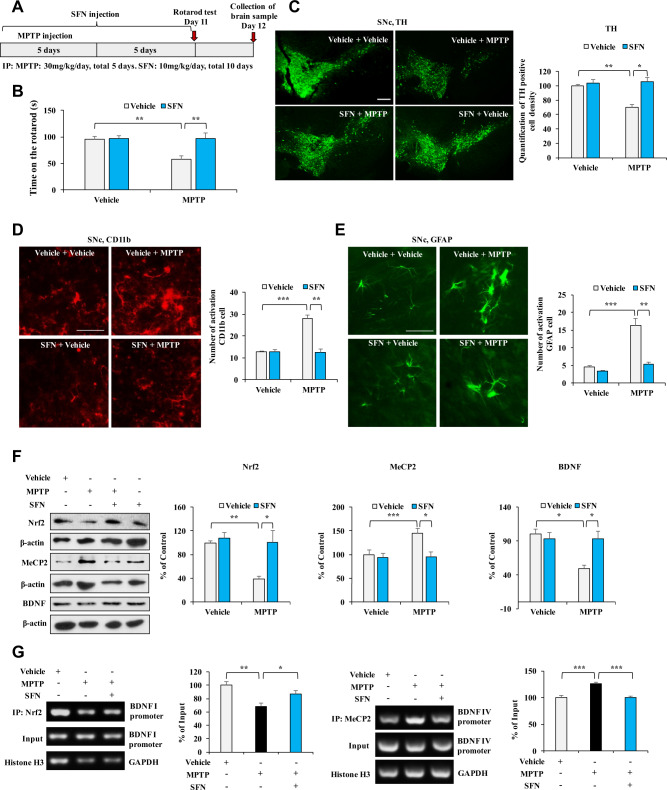
Effects of SFN on rotarod behavior and dopaminergic neurotoxicity in the SNc of MPTP-treated mice. **A** The schedule of treatments of MPTP and SFN. **B** The rotarod test showed that SFN attenuated MPTP-induced decreased of the rotarod time. Data are shown as mean ± SEM (*n* = 8 or 10). *P < 0.05, **P < 0.01 (two-way ANOVA: MPTP: *F*_1,31_ = 5.392, *P* = 0.028; SFN: *F*_1,31_ = 5.732, *P* = 0.024; interaction: *F*_1,31_ = 5.150, *P* = 0.031). **C** The immunofluorescence staining for TH in the SNc of mice treated with SFN or/and MPTP and quantification analysis of the TH staining in the SNc. Scale bar = 50 μm. Data are shown as mean ± SEM (*n* = 4). *P < 0.05, **P < 0.01 (two-way ANOVA: MPTP: *F*_1,15_ = 5.125, *P* = 0.043; SFN: *F*_1,15_ = 9.606, *P* = 0.009; interaction: *F*_1,15_ = 6.235, *P* = 0.028). **D** The immunofluorescence staining for CD11b in the SNc of mice treated with SFN or/and MPTP and quantification analysis of the CD11b staining in the SNc. Data are shown as mean ± SEM (*n* = 4). *P < 0.05, **P < 0.01 (two-way ANOVA: MPTP: *F*_1,15_ = 14.652, *P* = 0.002; SFN: *F*_1,15_ = 16.345, *P* = 0.002; interaction: *F*_1,15_ = 16.549, *P* = 0.002). Scale bar = 50 μm. **E** The immunofluorescence staining for GFAP in the SNc of mice treated with SFN or/and MPTP and quantification analysis of the GFAP staining in the SNc. Scale bar = 50 μm. Data are shown as mean ± SEM (*n* = 4). *P < 0.05, **P < 0.01 (two-way ANOVA: MPTP: *F*_1,15_ = 21.504, *P* = 0.001; SFN: *F*_1,15_ = 16.644, *P* = 0.002; interaction: *F*_1,15_ = 10.459, *P* = 0.007). **F** The effects of SFN on the expression of Nrf2, MeCP2, and BDNF in the SNc of mice treated with SFN or/and MPTP. Data are shown as mean ± SEM (*n* = 6). **P < 0.01 (two-way ANOVA: Nrf2, MPTP: *F*_1,23_ = 8.578, *P* = 0.008; SFN: *F*_1,23_ = 8.951, *P* = 0.007; interaction: *F*_1,23_ = 5.320, *P* = 0.032; MeCP2, MPTP: *F*_1,23_ = 5.342, *P* = 0.032; SFN: *F*_1,23_ = 8.166, *P* = 0.010; interaction: *F*_1,23_ = 4.903, *P* = 0.039; BDNF, MPTP: *F*_1,23_ = 8.610, *P* = 0.008; SFN: *F*_1,23_ = 4.591, *P* = 0.045; interaction: *F*_1,23_ = 8.548, *P* = 0.008). **G** The ChIP-PCR analysis in SFN or/and MPTP-treated mice. The binding affinity of SFN for Nrf2 and *Bdnf* I promoter and MeCP2 for *Bdnf* IV promoter in SNc of MPTP-treated mice. Data are shown as mean ± SEM (*n* = 4).　*P < 0.05, **P < 0.01, ***P < 0.001 (one-way ANOVA: Nrf2: *F*_2,14_ = 7.706, *P* = 0.007, MeCP2: *F*_2,14_ = 18.17, *P* < 0.01).

We examined whether SFN attenuates abnormal behavior and dopaminergic neurotoxicity in MPTP-treated mice (Fig. 3A). The behavioral study showed that MPTP (30 mg/kg/day for 5 days) significantly reduced the duration time for the mice on the rotarod test compared to those of the vehicle group, which could be reversed by SFN (10 mg/kg/day for 10 days) (Fig. 3B). SFN did not alter the duration time for the rotarod test in control mice (Fig. 3B). Next, we performed the immunofluorescent staining for tyrosine hydroxylase (TH), CD11b (for microglia) [34], and glial fibrillary acidic protein (GFAP for astrocyte) [35]. Immunofluorescent staining showed that MPTP significantly decreased TH-immunoreactivity and increased CD11b- and GFAP-immunoreactivity in the SNc, which could be reversed by SFN (Fig. 3C–E). In addition, we measured the protein levels of Nrf2, MeCP2, and BDNF in the SNc of MPTP-treated mice. We found the decreased expression of Nrf2 and BDNF, and increased expression of MeCP2 in the SNc of MPTP-treated mice compared to control mice (Fig. 3F). Furthermore, SFN significantly attenuated the alterations of these proteins in the SNc of MPTP-treated mice (Fig. 3F).

To confirm whether SFN can activate BDNF transcription in the SNc of MPTP-treated mice, we performed ChIP-PCR. We found that Nrf2 partially dissociated from *Bdnf* exon I promoter and that MeCP2 was more tightly associated with *Bdnf* exon IV promoter in the SNc of MPTP-treated mice (Fig. 3G). In addition, these changes were reversed by SFN (Fig. 3G). These results suggest that SFN can attenuate dopaminergic neurotoxicity and neuroinflammation in the SNc of MPTP-treated mice through activation of Nrf2 and BDNF and inhibition of MeCP2.

### Increased BDNF transcription via silencing MeCP2 expression by MeCP2-HDO

**Fig. 4:**
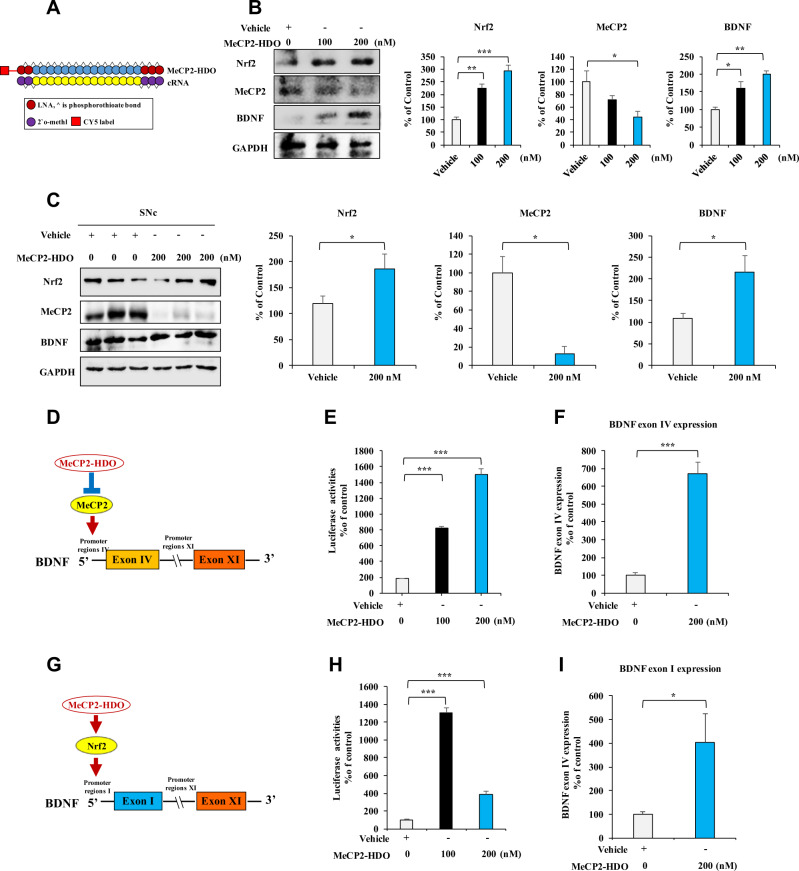
Effects of MeCP2-HDO on BDNF production through activation of BDNF transcription. **A** Schematic illustration of the construction of ASO, cRNA, and HDO. **B** MeCP2-HDO silenced MeCP2 and promoted Nrf2 and BDNF expression, in a dose-dependent manner. The SH-SY5Y cells were treated with various concentrations of MeCP2-HDO 24 h, and the cells sample was collected and analyzed by western blotting assay. Data are shown as mean ± SEM (*n* = 3). *P < 0.05, **P < 0.01 (one-way ANOVA: Nrf2: *F*_2,8_ = 37.650, *P* < 0.001, MeCP2: *F*_2,8_ = 5.351, *P* = 0.046, BDNF: *F*_2,8_ = 15.121, *P* = 0.005). **C** MeCP2-HDO silenced MeCP2 and promoted Nrf2 and BDNF expression in vivo. Mice were treated with MeCP2-HDO on day 1 and day 5. The levels of BDNF and MeCP2 in the SNc were examined by western blotting assay. Data are shown as mean ± SEM (*n* = 3). *P < 0.05 (Student *t* test: *T* = −2.782, *P* = 0.049 for Nrf2, *T* = 4.505, *P* = 0.011 for MeCP2, and *T* = −2.860, *P* = 0.046 for BDNF). **D** The schematic diagram of BDNF transcription by MeCP2-HDO. **E** Effects of MeCP2-HDO on the activation of *Bdnf* exon IV promoter by using luciferase reporter assay. Data are shown as mean ± SEM (*n* = 4). ***P < 0.001 (one-way ANOVA: *F*_2,14_ = 115.359, *P* < 0.001). **F** MeCP2-HDO increased *Bdnf* exon IV mRNA expression. Data are shown as mean ± SEM (*n* = 6). ***P < 0.001 (Student *t* test: *T* = −10.743, *P* < 0.001). **G** The schematic diagram of BDNF transcription via Nrf2 by MeCP2-HDO. **H** Effects of MeCP2-HDO on the activation of *Bdnf* exon I promoter by using luciferase reporter assay. Data are shown as mean ± SEM (*n* = 4). ***P < 0.001 (one-way ANOVA: *F*_2,14_ = 279.849, *P* < 0.001). **I** MeCP2-HDO increased *Bdnf* exon I mRNA expressions. Data are shown as mean ± SEM (*n* = 4 or 5). *P < 0.05 (Student *t* test: *T* = −2.494, *P* = 0.041).

The aforementioned data suggest that silenced MeCP2 expression may ameliorate dopaminergic neurotoxicity in the SNc of MPTP-treated mice. To address this hypothesis, we knocked down MeCP2 expression with a newly designed MeCP2-HDO which has been demonstrated as a potent silencing technique with high efficacy in the brain [29, 30]. As shown in Fig. 4A, a DNA and RNA heteroduplex oligonucleotide was designed to target MeCP2. In SH-SY5Y cell, MeCP2-HDO significantly decreased MeCP2 and increased Nrf2 and BDNF expressions, in a dose-dependent manner (Fig. 4B). In vivo, MeCP2-HDO downregulated MeCP2 and upregulated Nrf2 and BDNF expressions in wild-type mice by ICV injection on day 1 and day 5 (Fig. 4C). Since MeCP2 functions as a transcription repressor of BDNF via motif ahead of *Bdnf* exon IV [23, 24], the effects of MeCP2-HDO for BDNF transcription were examined by luciferase reporter assay. MeCP2-HDO activated BDNF exon IV promoter, in a dose-dependent manner (Fig. 4D, E). Furthermore, the levels of *Bdnf* exon IV mRNA were significantly upregulated by MeCP2-HDO (Fig. 4F). Collectively, these data suggest that downregulation of MeCP2 could promote Nrf2 expression and BDNF expression. To confirm this hypothesis, we examined the BDNF exon I promoter activity by luciferase reporter assay after administration of MeCP2-HDO. MeCP2-HDO significantly activated BDNF exon I promoter at low and high doses (Fig. 4G, H). Furthermore, the levels of *Bdnf* exon I mRNA were upregulated by MeCP2-HDO (Fig. 4I). These results suggest that MeCP2-HDO effectively silences MeCP2 in both in vitro and in vivo, leading to BDNF transcription.

### Effects of MeCP2-HDO on dopaminergic neurotoxicity in MPTP-treated mice

**Fig. 5:**
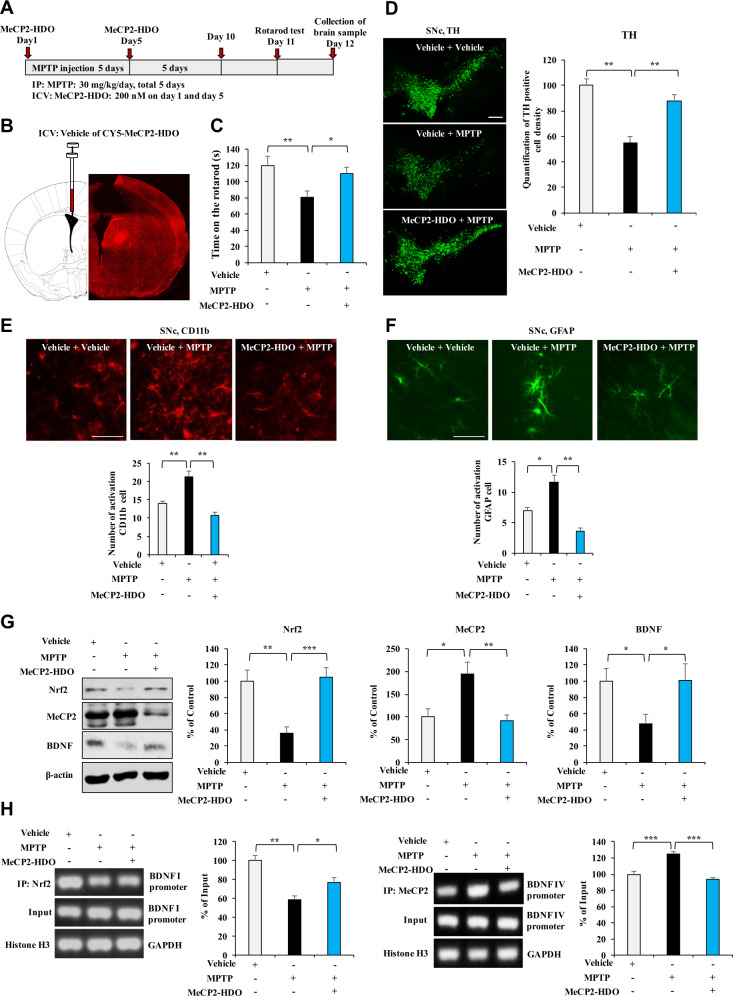
Effects of MeCP2-HDO on dopaminergic neurotoxicity in MPTP-treated mice. **A** Schedule of treatment, behavioral test and collection of samples. **B** The schematic diagram of the ICV injection of MeCP2-HDO in the brain. **C** MeCP2-HDO reversed the decreased time of rotarod time in MPTP-treated mice. Data are shown as mean ± SEM (*n* = 10). *P < 0.05; **P < 0.01 (one-way ANOVA: *F*_2,29_ = 4.452, *P* = 0.021). **D** The immunofluorescence staining for TH in the SNc of the mice treated with MeCP2-HDO or/and MPTP and quantification of the TH staining. Data are shown as mean ± SEM (*n* = 4). **P < 0.01 (one-way ANOVA: *F*_2,11_ = 14.083, *P* = 0.002). Scale bar = 50 μm. **E** The immunofluorescence staining for CD11b in the SNc of the mice treated with MeCP2-HDO or/and MPTP and quantification of the CD11b staining. Data are shown as mean ± SEM (*n* = 4). **P < 0.01 (one-way ANOVA: *F*_2,11_ = 13.573, *P* = 0.002). Scale bar = 50 μm. **F** The immunofluorescence staining for GFAP in the SNc of the mice treated with MeCP2-HDO or/and MPTP and quantification of the GFAP staining. Data are shown as mean ± SEM (*n* = 4). **P < 0.01 (one-way ANOVA: *F*_2,11_ = 12.309, *P* = 0.003). Scale bar = 50 μm. **G** Effects of MeCP2-HDO on the levels of Nrf2, MeCP2, and BDNF expression in the SNc of MPTP-treated mice. Data are shown as mean ± SEM (*n* = 7 or 8). *P < 0.05, **P < 0.01, ***P < 0.001 (one-way ANOVA: Nrf2: *F*_2,23_ = 11.961, *P* < 0.001, MeCP2: *F*_2,22_ = 7.717, *P* = 0.003, BDNF: *F*_2,23_ = 3.674, *P* = 0.043). **H** The ChIP-PCR analysis in the MeCP2-HDO or/and MPTP-treated mice. The binding affinity of MeCP2-HDO for Nrf2 and *Bdnf* I promoter and MeCP2 for *Bdnf* IV promoter in the SNc of MPTP-treated mice. Data are shown as mean ± SEM (*n* = 4). *P < 0.05, **P < 0.01, ***P < 0.001 (one-way ANOVA: Nrf2: *F*_2,11_ = 13.680, *P* = 0.002, MeCP2: *F*_2,11_ = 26.494, *P* < 0.001).

We examined the effects of MeCP2-HDO on MPTP-induced dopaminergic neurotoxicity in the mouse brain (Fig. 5A). As shown in Fig. 5B, CY5-tagged MeCP2-HDO was distributed widely in the mouse brain. In the rotarod test, MPTP significantly reduced the duration time for the mice on the rotarod test compared to those of the vehicle group, which were reversed by ICV injection of MeCP2-HDO (200 nM on day 1 and day 5) (Fig. 5C). Immunofluorescent staining demonstrated that MeCP2-HDO significantly ameliorated the reduced TH-immunoreactivity in the SNc (Fig. 5D), and the increased CD11b- and GFAP-immunoreactivity in SNc of MPTP-treated mice (Fig. 5E, F). Western blot analysis showed that MeCP2-HDO ameliorated abnormal expression of Nrf2, MeCP2, and BDNF in the SNc of MPTP-treated mice (Fig. 5G).

To further confirm whether MeCP2-HDO can activate BDNF transcription in SNc of MPTP-treated mice, we performed ChIP-PCR. Nrf2 partially dissociated from *Bdnf* exon I promoter and MeCP2 was more tightly associated with *Bdnf* exon IV promoter in SNc of MPTP-treated mice (Fig. 5H). Furthermore, these changes by MPTP treatment were significantly reversed by MeCP2-HDO (Fig. 5H).

### Decreased expressions of Nrf2, BDNF, and increased expression of MeCP2 in the striatum from DLB patients

**Fig. 6:**
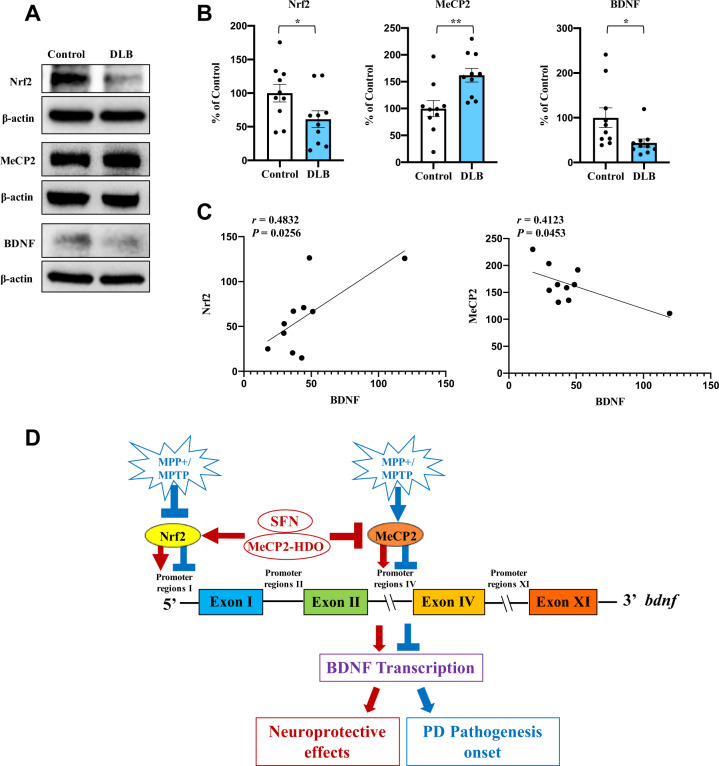
The protein levels of Nrf2, BDNF, and MeCP2 in the striatum of DLB patients and the working model for this study. **A**, **B** Protein expression of Nrf2, MeCP2 and BDNF in the striatum from DLB patients (*n* = 10) and controls (*n* = 10). Representative immunoblots were shown from the two groups. Data are shown as mean ± SEM (*n* = 10). *P < 0.05, **P < 0.01 (Student *t* test: *T* = −2.138, *P* = 0.046 for Nrf2, *T* = −3.448, *P* = 0.003 for MeCP2, and *T* = 2.361, *P* = 0.030 for BDNF). **C** There was a positive correlation between BDNF levels and Nrf2 levels in the subjects (*n* = 10). Furthermore, there was a negative correlation between BDNF levels and MeCP2 levels in the subjects (*n* = 10). **D** MPP^+^ and MPTP inhibit Nrf2 and upregulate MeCP2 expressions, which leads to suppress BDNF transcription and promotes dopaminergic neurotoxicity. SFN and MeCP2-HDO induce BDNF transcription by activating *Bdnf* exon I promoter and *Bdnf* exon IV promoter, resulting in neuroprotective effects against PD pathogenesis.

To confirm whether expression of Nrf2, MeCP2, and BDNF in the striatum are altered in patients of PD, we used postmortem brain samples from patients with DLB, since the postmortem pathological changes of DLB patients are similar to PD patients [36]. We measured the protein expression of Nrf2, BDNF, and MeCP2 in the striatum from DLB patients (*n* = 10) and age-matched control subjects (*n* = 10). Protein levels of Nrf2 and BDNF in the striatum from DLB patients were significantly lower than those of the controls, whereas protein levels of MeCP2 in the striatum from DLB patients were significantly higher than those of controls (Fig. 6A, B). Interestingly, there was a positive correlation between BDNF levels and Nrf2 levels in DLB patients (Fig. 6C). Furthermore, there was a negative correlation between BDNF levels and MeCP2 levels in DLB patients (Fig. 6C). Collectively, it is likely that both decreased Nrf2 and increased MeCP2 expressions are associated with decreased BDNF expression, suggesting that abnormalities of these signaling might play a role in the pathogenesis of PD.

## Discussion

The major findings of the present study are as follows. First, SFN could activate BDNF transcription through Nrf2 binding with the *Bdnf* exon I promotor. Furthermore, SFN could ameliorate the downregulation of Nrf2 and BDNF, and upregulation of MeCP2 in MPP^+^-treated SH-SY5Y cells. Second, SFN attenuated behavioral abnormality and dopaminergic neurotoxicity in the SNc of MPTP-treated mice. Furthermore, SFN attenuated the decreased expression of Nrf2 and BDNF, and the increased expression of MeCP2 in the SNc of MPTP-treated mice. Third, MeCP2-HDO decreased MeCP2 expression, as well as increased Nrf2 and BDNF expression in SH-SY5Y cells. Furthermore, ICV injection of MeCP2-HDO improved behavioral abnormality and dopaminergic neurotoxicity in the SNc of MPTP-treated mice. In addition, MeCP2-HDO improved abnormal expressions of Nrf2, MeCP2 and BDNF in the SNc of MPTP-treated mice. Finally, the protein expression of Nrf2 and BDNF was lower in the striatum of DLB patients than that of controls, whereas the protein expression of MeCP2 was higher in the striatum of DLB patients than that of controls. Interestingly, there were correlations between BDNF and Nrf2 (or MeCP2) in the striatum from the DLB. Therefore, it is likely that the activation of BDNF transcription by activation of Nrf2 and/or suppression of MeCP2 could be a new therapeutic approach for PD.

As aforementioned, BDNF and its receptor TrkB are known to co-localize with dopaminergic neurons in the SNc, and BDNF promotes dopaminergic neuronal survival [3, 37]. It is reported that overexpression of BDNF could attenuate 6-OHDA or MPTP-induced nigrostriatal degeneration and rotational behavior defect [11, 12]. Interestingly, 7,8-dihydroxyflavone, a potent TrkB agonist, demonstrated to protect against MPTP-induced dopaminergic neurotoxicity in rodents and monkeys [38, 39]. It is also reported that (*R*)-ketamine attenuated MPTP-induced dopaminergic neurotoxicity in the mouse striatum through activation of the TrkB signaling pathway [40]. In this study, we found that activation of BDNF transcription through activation of Nrf2 by SFN contributes to its protective effects for MPTP-induced neurotoxicity. Collectively, it seems that activation of the BDNF-TrkB signaling pathway in the brain could produce protective effects for dopaminergic neurotoxicity in the brain of PD.

As a potent Nrf2 activator, SFN is well known to promote phase II detoxification enzymes and antioxidant protein transcription [17, 32, 41–43]. SFN glucosinolate, a precursor of SFN, is found in cruciferous vegetables such as broccoli, brussels sprouts, and cabbage [15, 21, 22], and it converts to SFN through the catalytic actions of plant myrosinase or beta-thioglucosidases in the gut microflora [44]. In this study, we found that SFN could ameliorate abnormal behavior and dopaminergic neurotoxicity in MPTP-treated mice through activation of BDNF. A previous study showed that SFN protected against MPTP-induced neurotoxicity in the wild-type mice, but not *Nrf2* KO mice, suggesting the role of Nrf2 in the protective effects of SFN [45]. A recent study showed that dietary intake of SFN glucosinolate could prevent MPTP-induced dopaminergic neurotoxicity in the mouse striatum [46]. Collectively, dietary intake of cruciferous vegetables including SFN glucosinolate might have a prophylactic effect for PD. A randomized, placebo-controlled study using vegetables including SFN glucosinolate in early phase patients with PD or prodromal phase subjects of PD is of great interest.

In this study, we found increased expression of MeCP2 in the MPP^+^-treated SH-SY5Y cells and in the SNc of MPTP-treated mice, suggesting a role of MeCP2 in PD pathogenesis. A study using MeCP2-HDO showed that dopaminergic neurotoxicity in the SNc of MPTP-treated mice could be ameliorated by upregulation of BDNF expression. Furthermore, we found higher expression of MeCP2 in the striatum from DLB patients compared to controls. It is shown that patients with mutations in the *MeCP2* exhibit motor deficits similar to PD patients, suggesting that MeCP2 may be involved in the pathogenesis of PD [27, 28]. Given the transcriptional regulation of BDNF by MeCP2 [25, 47–49], it is possible that MeCP2 would be a new therapeutic target for PD and its related neurodegenerative disorders such as DLB. It seems that small compounds such as AG490 would be potential therapeutic drugs for PD since AG490 can reactivate MeCP2 [50].

A recent study shows that Nrf2 can bind to BDNF exon I promoter, resulting in BDNF transcription [25, 51]. Furthermore, MeCP2 is a transcription repressor, which blocks BDNF transcription [23, 24]. Therefore, Nrf2 and MeCP2 might bidirectionally regulates BDNF transcription. In this study, we found that activation of Nrf2 by SFN is associated with *Bdnf* exon I promoter and that MeCP2 is more dissociated with *Bdnf* exon IV promoter in the SNc of MPTP-treated mice. These findings suggest a possibility that increased Nrf2 or decreased MeCP2 expression may contribute to BDNF transcription, which plays a role in the treatment of PD.

In conclusion, this study suggests that SFN could protect against MPTP-induced dopaminergic neurotoxicity in the SNc by activation of BDNF as well as by inhibition of MeCP2. Therefore, it is likely that MeCP2 could be a new therapeutic target for neurodegenerative disorders such as PD.

## Materials and methods

### Mice and cell lines

Male adult C57BL/6 mice (8 weeks old, 20–25 g each, Guangdong Experimental Animal Center, China) were used in experiments. Age-matched animals were randomly allocated to experimental groups. The sample sizes were based on previous experience with the experimental design [1, 3, 33]. Since several batched of mice were tested independently and pooled together for final analyses. Therefore, the group sizes are not exactly the same. The criteria were not pre-established. The animals were housed under controlled temperature and kept on a 12-h light/dark cycle (lights on between 07:00 and 19:00), with ad libitum access to food and water. The protocol was approved by the Jinan University Institutional Animal Care and Use Committee. All experiments were carried out following the Guide for Animal Experimentation of Jinan University. SH-SY5Y human neuroblastoma cell line was cultured in DMEM/F-12 (BasaIMedia) supplemented with 10% fetal bovine serum (Excell Bio.) and penicillin (100 units/mL)–streptomycin (100 μg/mL) (Hyclone). HEK293T cells were maintained in DMEM supplemented with FBS and penicillin–streptomycin as described above. Cells were cultured at 37 °C in a humidified incubator containing 5% CO_2_. All the cell lines were tested for mycoplasma contamination.

### Reagents and drug treatment

MPP^+^ and MPTP were purchased from MedChemExpress (Monmouth Junction, NJ, USA), which were dissolved in phosphate-buffered saline (PBS) or physiological saline. SFN (LKT Laboratories, Inc., St Paul, MN, USA) was dissolved in distilled water containing 10% corn oil. SFN (1 μM) and/or MPP^+^ (1 mM) were treated for SH-Y5Y cells. SFN (10 mg/kg) and MPTP (30 mg/kg) was administered intraperitoneally (i.p.) to mice. The concentration and dosages of MPP^+^, SFN, and MPTP were selected as previously reported [3, 22, 33].

### MeCP2-HDO construction

The antisense oligonucleotides (ASO) of MeCP2 and cRNA were designed and purchased from TsingKe biological technology (Wuhan, China). For the generation of MeCP2-HDO, equimolar amounts of DNA and cRNA strands were heated in 0.9% sterile saline at 95 °C for 5 min and slowly cooled to room temperature. HDO that carried locked nucleic acids (LNA) at each end flanking the central base of DNA and with or without CY5 label, and carried 2’-O-methyl at each end flanking the central base of cRNA. The sequences of ASOs and cRNA targeting MeCP2 used in our experiments were listed below: ASO-MeCP2: G(L)^G(L)^t^t^t^t^t^c^t^c^c^t^t^t^a^t^t^A(L)^T(L)^C(L) [52]; cRNA: g(M)^a(M)^u(M)^aauaaaggagaaaaa^c(M)^c(M). L stands for locked nucleic acids. M stands for 2’-O-methyl modifications. ^ stands for phosphorothioate bond.

### Intracerebroventricular (ICV) injection

Mice were anesthetized with isoflurane and fixed to the stereotaxic apparatus. Drugs were injected into the right lateral ventricle following the stereotaxic coordinates: 0.8 mm lateral, −2.1 mm ventral, and 0.74 mm from Bregma. For injection two times, a guiding cannula (RWD Life Science, China) was implanted using the coordinates as described above. The concentration of MeCP2-HDO was 200 nM. The volume was 2 μl per injection. Drugs were injected by an injection cannula through a guiding cannula.

### The rotarod test

Mice were first trained to stay on the rod of the rotarod, which was maintained at a constant speed (5 rpm) for at least 5 min. After training for three times, mice were tested for a total of three trials with a constant rotation speed from 0 rpm to a maximum 40 rpm. The trial was started and sustained for 5 min, then stopped when the mouse fell (activating a switch that automatically stopped the timer) or when 5 min had elapsed. The residence time on the rotarod was counted using the stopwatch by a researcher blind to the treatment.

### Quantitative real-time PCR assay (qRT-PCR)

Levels of *Bdnf* exon I promoter and *Bdnf* exon IV promoter mRNA were examined by quantitative real-time PCR. For cDNA collection, RNA was extracted by using an Eastep^®^ Super Kit (Promega) followed by reverse transcription with GoScript^TM^ Reverse Transcriptase Mix, Oligo (dT) (Promega). All real-time PCRs were performed with the ChamQ^TM^ SYBR^®^ qPCR Master Mix Kit (Vazyme) in the 788BR05175 Real-Time PCR System. Forty cycles of PCR amplification were performed as follows: denaturation at 95 °C for 30 s, annealing at 55 °C for 30 s, and extension for 30 s at 72 °C. The primer sequences were as follows: forward 5’-TGATCATCACTCACGACCACG-3’; reverse 5’ CAGCCTCTCTGAGCCAGTTACG-3’ for *Bdnf* exon I; forward 5’-GGCTTCTGTGTGCGTGAATTTGC-3’; reverse 5’-AAAGTGGGTGGGAGTCCACGAG-3’ for *Bdnf* exon IV; forward 5’ ATGACATCAAGAAGGTGGTG-3’; reverse 5’-CATACCAGGAAATGAGCTTG-3’ for *gapdh*. The target genes were analyzed by the 2^−ΔΔCt^ method.

### Luciferase reporter assay

HEK293T cells were seeded in 6-wells plates and transfected/treated with BDNF exon I or BDNF exon IV luciferase reporter plasmid, pRL-TK Renilla luciferase plasmid (Promega), and SFN. Following treatment, the cells were collected and subjected to the dual-luciferase reporter assay kit (Promega) according to the manual.

### Chromatin immunoprecipitation (ChIP) assay

Brain samples were subjected to the ChIP assay according to the manual of the SimpleChIP^®^ Enzymatic Chromatin IP Kit (Cell Signaling). In the ChIP assay, 7.5 μg of Nrf2 or p-CREB antibody was added to the homogenate of the samples, mixed, and incubated overnight at 4 °C. Washing, elution, and reverse cross-linking to free DNA were performed according to the manufacturer’s protocol. *Bdnf* exon I or *Bdnf* exon IV-specific primers were used for amplification of the promoter region. The primer sequences were as follows: forward *Bdnf* exon I: 5’-TGATCATCACTCACGACCACG-3’; reverse 5’-CAGCCTCTCTGAGCCAGTTACG-3’. forward *Bdnf* exon IV: 5’-GGCTTCTGTGTGCGTGAATTTGC-3’; reverse 5’-AAAGTGGGTGGGAGTCCACGAG-3’. The PCR amplicon was separated on a 2% agarose gel after 35 cycles of PCR (denaturation at 95 °C for 30 s, annealing at 58 °C for 30 s, and extension at 72 °C for 30 s).

### Western blot analysis

Cells and brain homogenates were lysed in RIPA buffer. Postmortem brain samples (striatum) from patients with DLB (*n* = 10) and age-matched controls (*n* = 10) were collected at Tokyo Metropolitan Geriatric Hospital and Institute of Gerontology (Tokyo, Japan), as previously reported [36]. Brain samples were selected using the Brain Bank for Aging Research (BBAR) Lewy bodies rating system [53]. Protein concentration was determined by the Coomassie Brilliant Blue protein assay kit (Bio-Rad). Twenty micrograms of total proteins were separated by 10–12% sodium dodecyl sulfate-polyacrylamide gels and then transferred to polyvinylidenedifluoride (PVDF) membrane. The membranes were blocked with 5% milk at room temperature for 1 h. The primary antibodies were added and incubated with the membranes at 4 °C for 12 h. Membranes were then washed three times with TBST and incubated with the corresponding secondary antibodies for 1 h at room temperature. After an additional three times of washing in TBST for 10 min, targeted protein bands were detected using the enhanced chemiluminescence method detected by the Tanon-5200CE imaging system (Tanon, Shanghai, China). The expression levels of target proteins were normalized to β-actin or GAPDH loading controls. Antibodies against Nrf2 (ab137550), BDNF (ab108319), and MeCP2 (ab2828) were purchased from Abcam, BDNF (47808) was purchased from Cell Signaling Technology (CST), MeCP2 (M6818) was purchased from Sigma-Aldrich. The β-actin or GAPDH antibody was purchased from EarthOx. The HRP-conjugated anti-rabbit IgG antibody and anti-mouse IgG antibody were purchased from BIO-RAD.

### Immunofluorescence

Cells or mouse brain sections were pre-planted on cover glasses and fixed in 4% PFA for 10 min at room temperature. After treatment, the glasses were washed with PBS three times and blocked with 3% BSA and 0.3% Triton X-100 for 30 min followed by incubation with anti-TH (1:500, GeneTex (GTX)10372), anti-CD11b (1:500, 2151423, Invitrogen) or anti-GFAP (1:500, Affitify, DF6040) for 24 h at 4 °C, respectively. After being washed with PBS, the cells were incubated with Alexa Fluor 488/594anti-mouse/Rabbit secondary antibody (1:500) for 2 h at room temperature in the dark followed by staining with DAPI to illustrate the nuclei. Then cells were washed in PBS and visualized by a fluorescence microscope (Olympus BX53, Japan).

### Statistical analysis

All data results were expressed as the mean ± standard error of the mean (SEM), which were analyzed using PASW Statistics 20 software. Potential differences between the mean values were evaluated using one-way analysis of variance (ANOVA), followed by post hoc Fisher’s least significant difference test or two-way ANOVA; when appropriate, post hoc comparisons were performed using the unpaired *t* test. *P* values <0.05 were considered statistically significant. Student’s *t* test was used to compare the differences between two groups, unless otherwise specified. Asterisks were used to indicate significance: *P < 0.05, **P < 0.01, and ***P < 0.001. Values >0.05 were considered not significant (ns).

## Supplementary information

Original Data File

## Data Availability

The datasets used and/or analyzed during this study are available from the corresponding author on reasonable request.
