# Supplementary figures and images for: Regulation of BDNF transcription by Nrf2 and MeCP2 ameliorates MPTP-induced neurotoxicity
Source: Cell Death Discov. 2022 May 20;8:267. doi: 10.1038/s41420-022-01063-9 (PMC9122988; doi:10.1038/s41420-022-01063-9)

Figure 1E

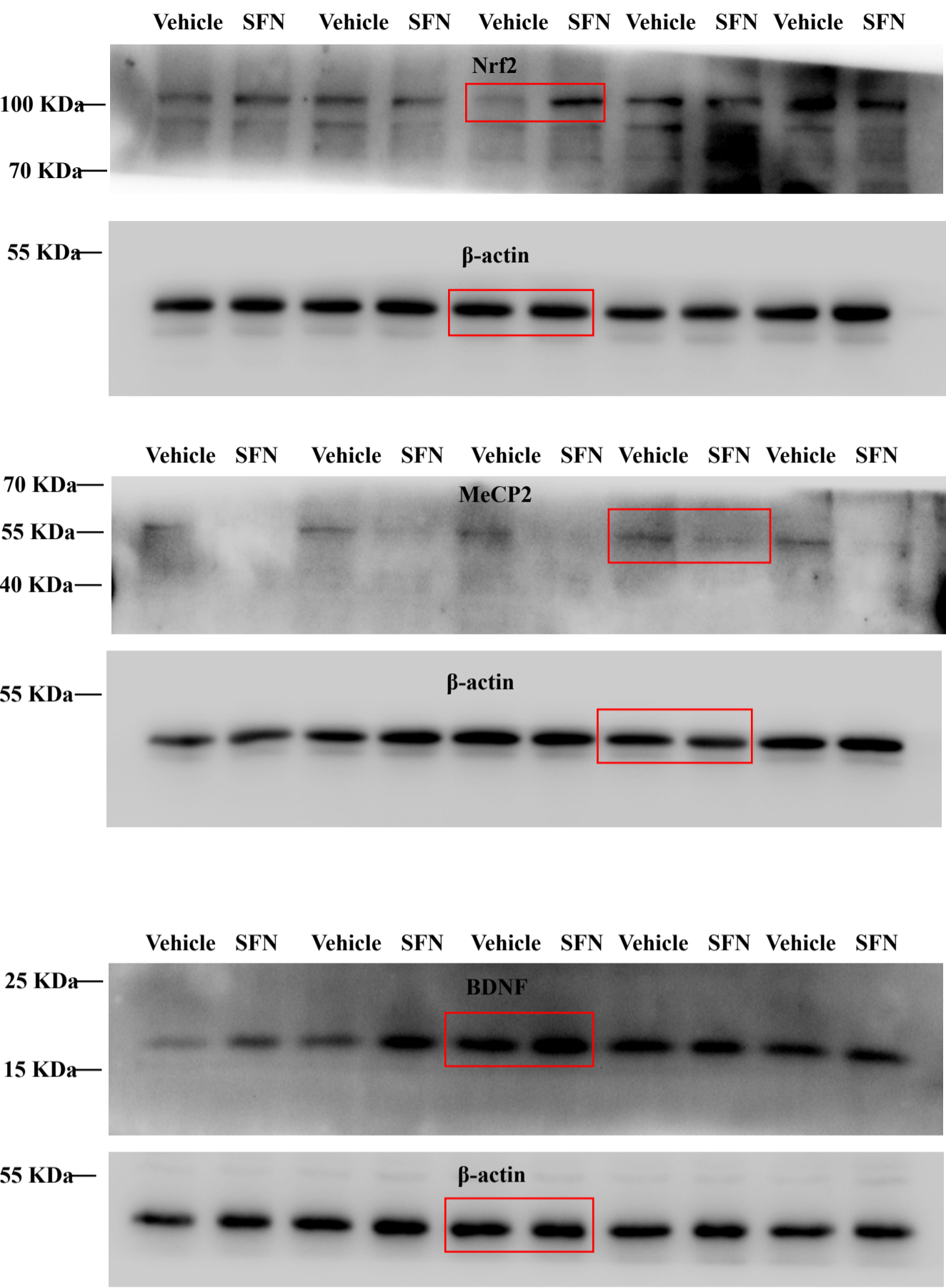

Figure 2A

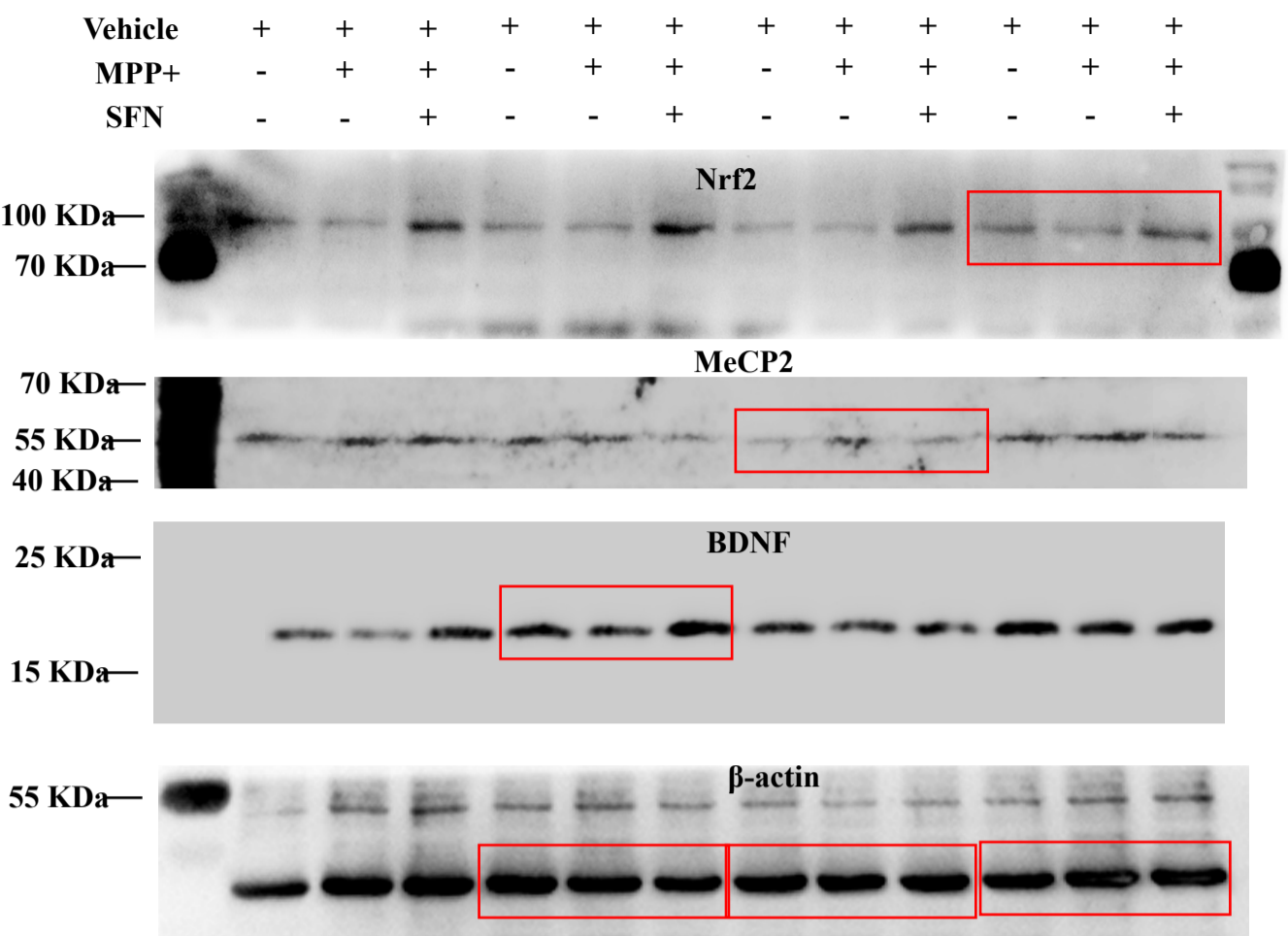

Figure 3F

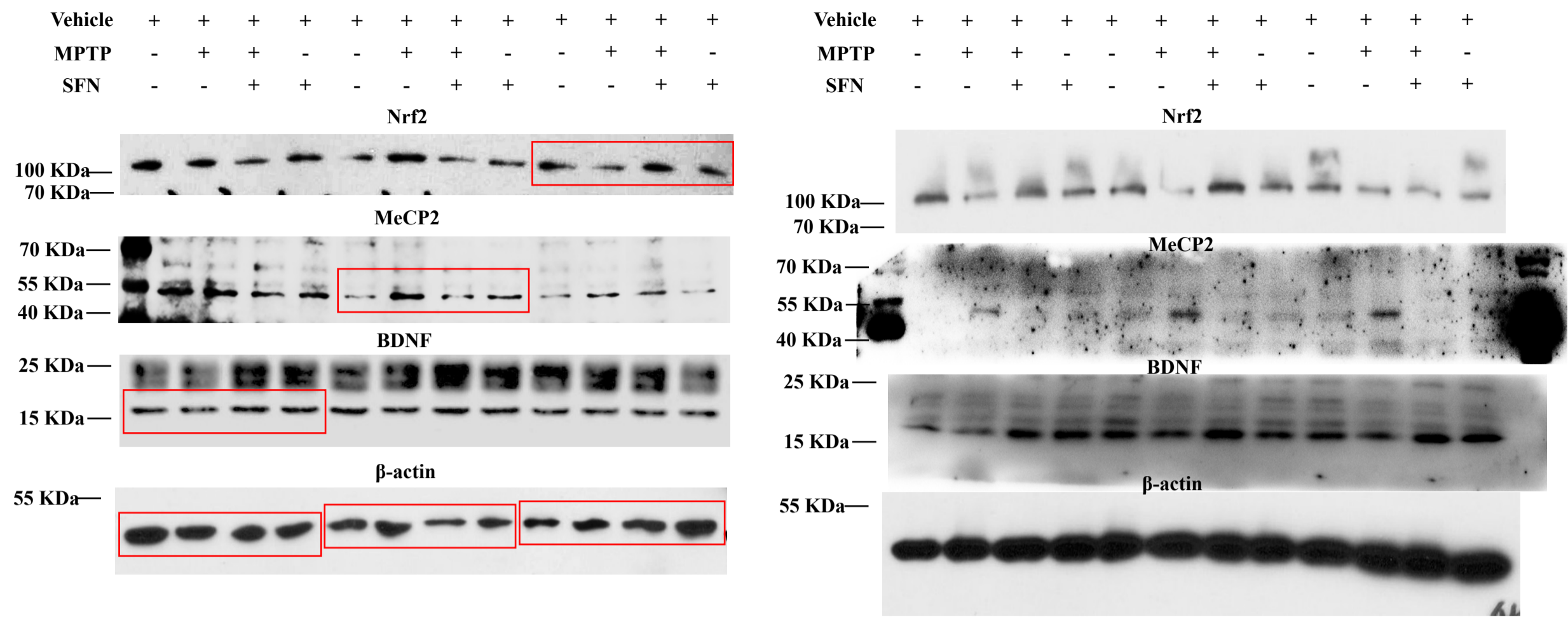

Figure 3G

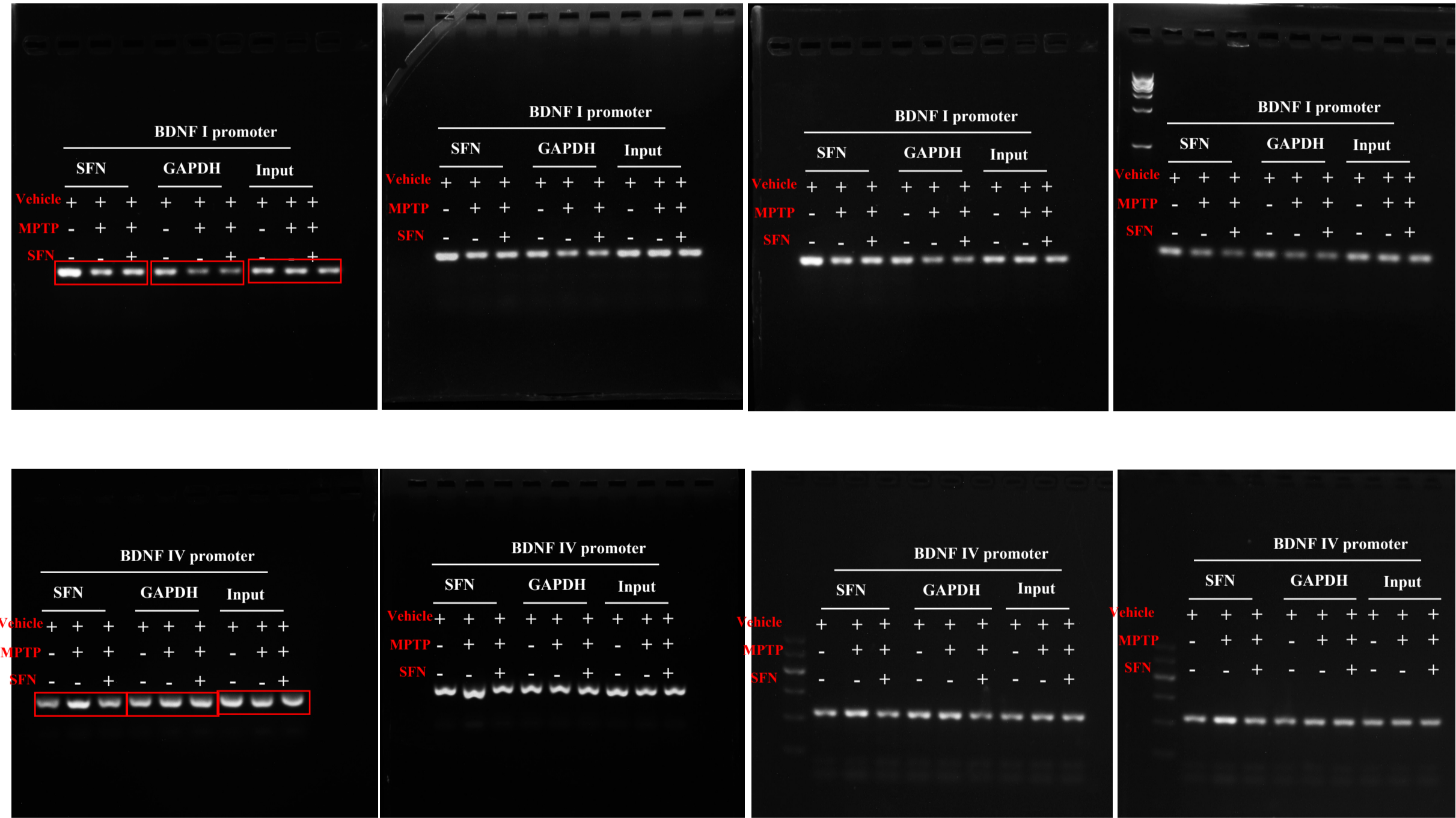

Figure 4B

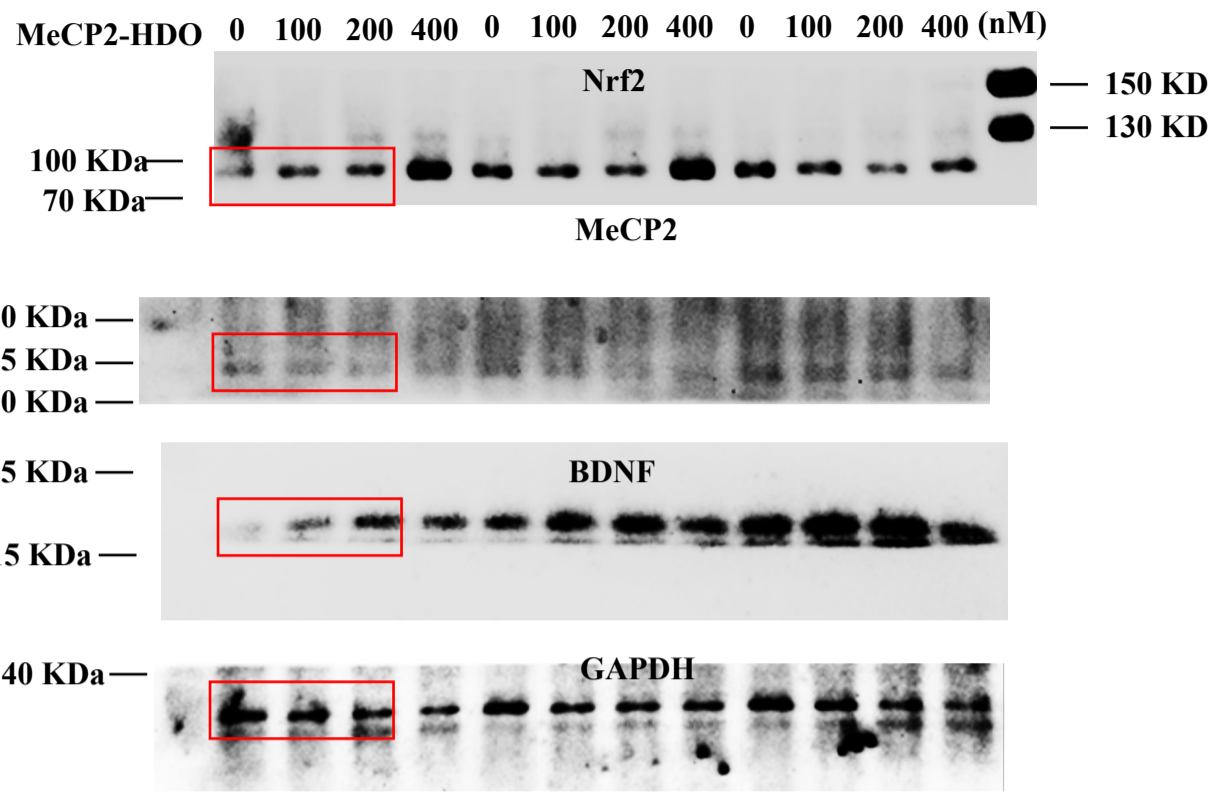

Figure 4C

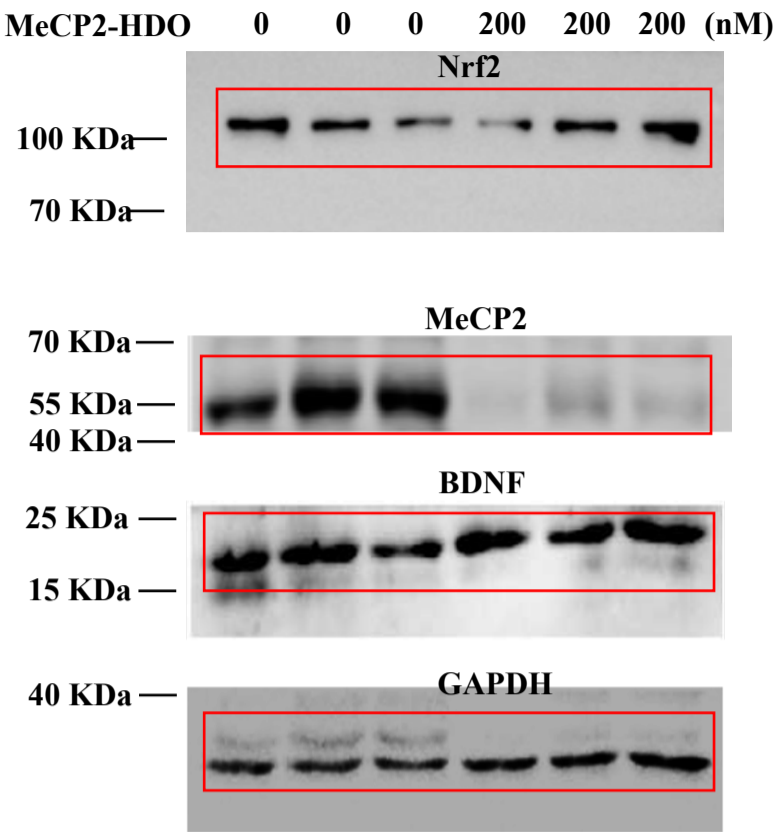

Figure 5G

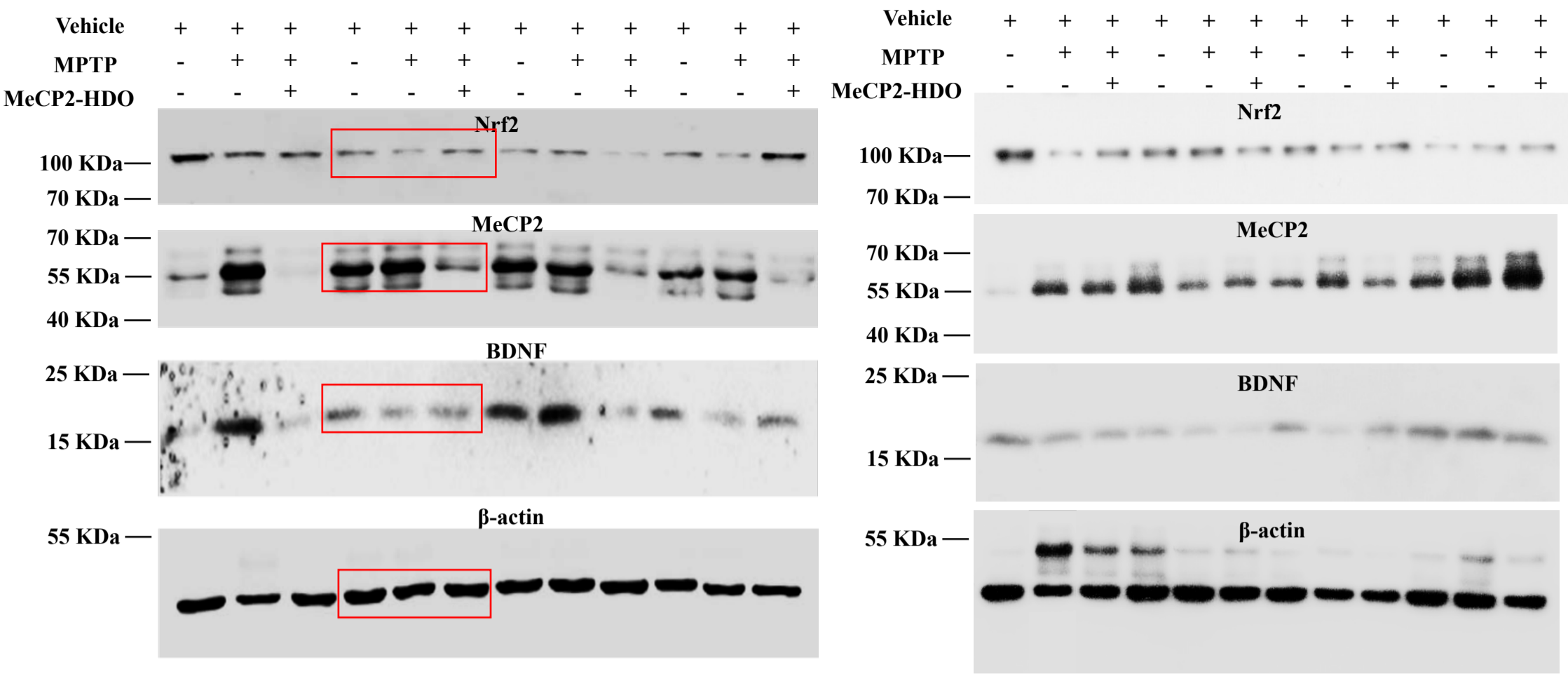

Figure 5H

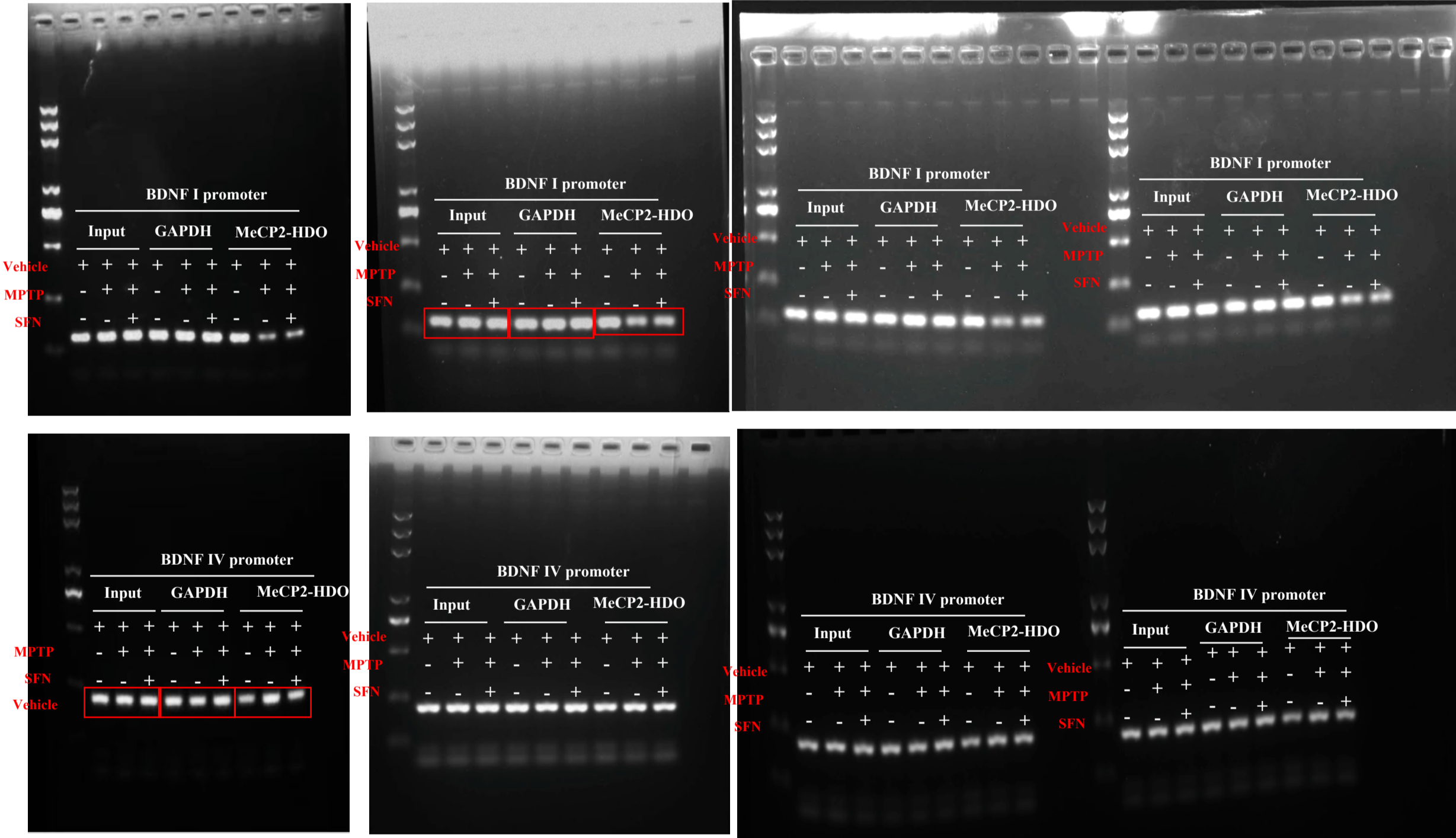

Figure 6A

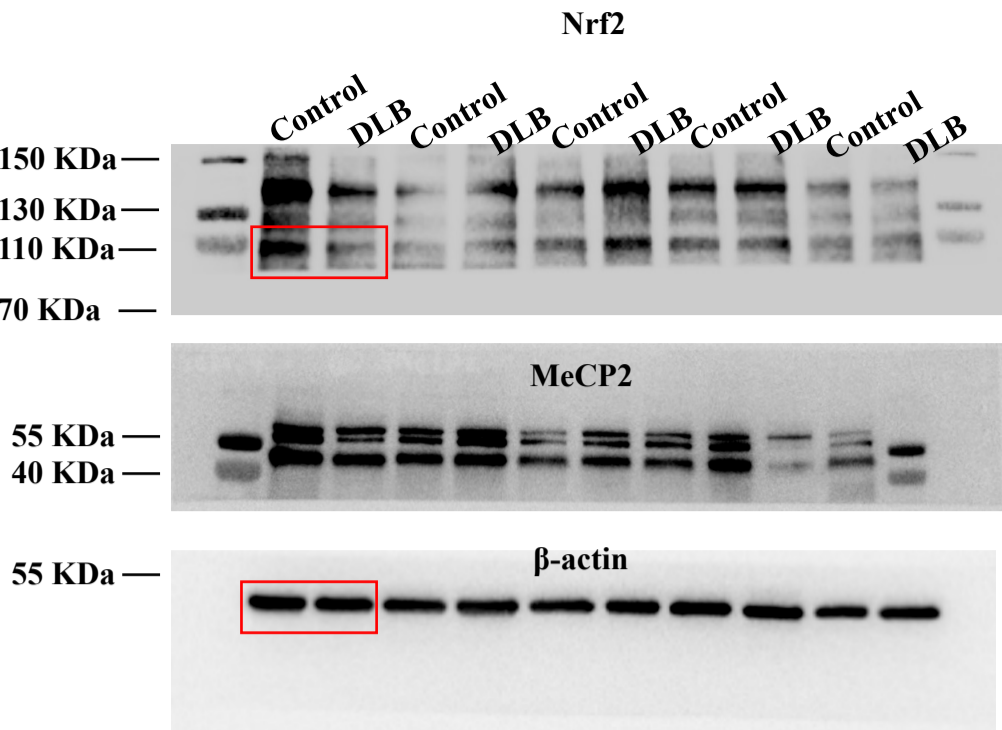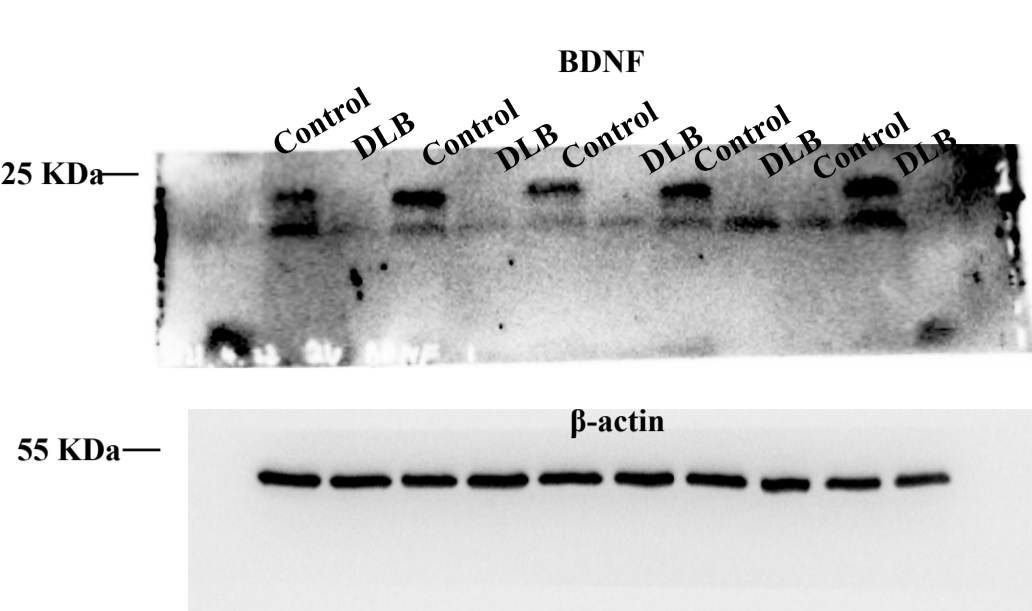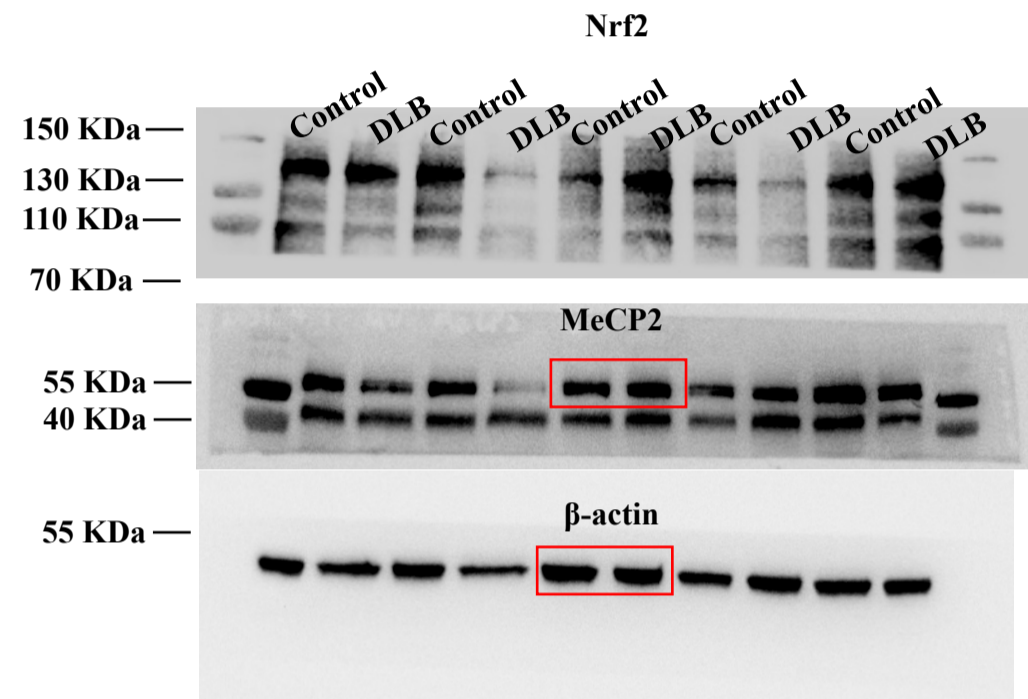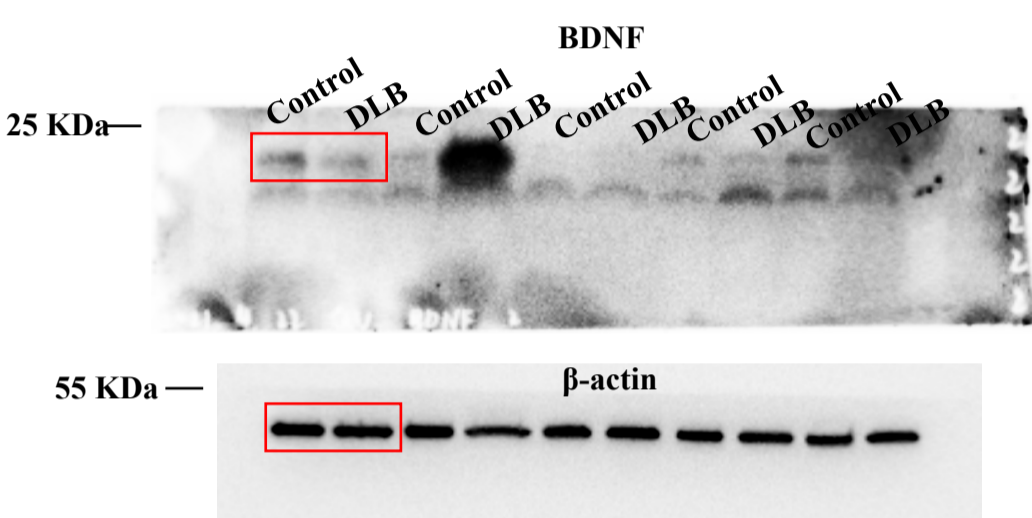

Supplement: Supplementary file 1 — Original Data File [file 41420_2022_1063_MOESM1_ESM.pdf]
